# Visual Servo Control System of a Piezoelectric2-Degree-of-Freedom Nano-Stepping Motor

**DOI:** 10.3390/mi10120811

**Published:** 2019-11-25

**Authors:** Cheng-Lung Chen, Shao-Kang Hung

**Affiliations:** Department of Mechanical Engineering, National Chiao Tung University, No. 1001, University Road, Hsinchu 30010, Taiwan; bruce.me08g@nctu.edu.tw

**Keywords:** piezoelectricity, visual servo control, stepping motor, nano-positioner, stick-slip

## Abstract

A nano-stepping motor can translate or rotate when its piezoelectric element pair is electrically driven in-phase or anti-phase. It offers millimeter-level stroke, sub-micron-level stepping size, and sub-nanometer-level scanning resolution. This article proposes a visual servo system to control the nano-stepping motor, since its stepping size is not consistent due to changing contact friction, using a custom built microscopic instrument and image recognition software. Three kinds of trajectories—straight lines, circles, and pentagrams—are performed successfully. The smallest straightness and roundness ever tested are 0.291 µm and 2.380 µm. Experimental results show that the proposed controller can effectively compensate for the error and precisely navigate the rotor along a desired trajectory.

## 1. Introduction

**Figure 1:**
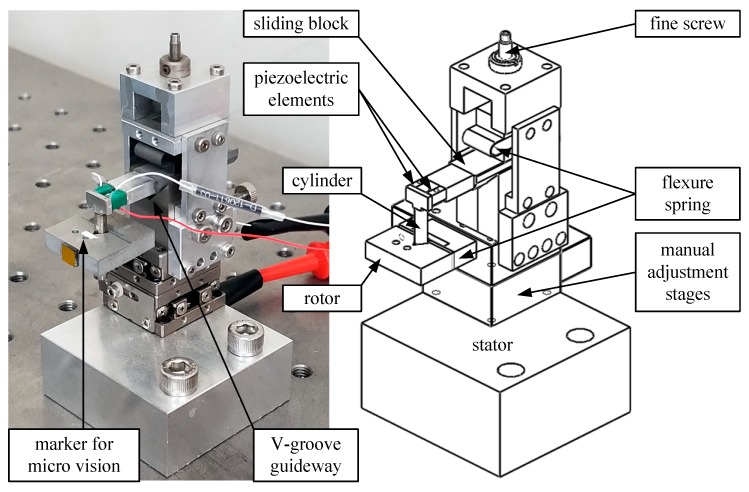
Photograph and schematic of a piezoelectric 2-DOF (degree of freedom) nano-stepping motor, which has a compact size of 5 cm × 5 cm × 3 cm. The fine screw presses the flexure spring and adjusts the clamping force between the V-groove guideway and the sliding block. The rotor can rotate around the cylinder. Translational or rotational stepping motions are generated when the pair of piezoelectric elements are driven in-phase or anti-phase, respectively.

A piezoelectric nano-stepping motor [1] was presented in our previous research work. This article presents its latest enhancements. The device has two degree-of-freedoms (DOF), translation for 6 mm and rotation for endless 360°. Its stepping resolution of translation and rotation are 100 nm and 0.04°, respectively. As it is well-known, piezoelectric elements have inherent nanometer resolution if they work in scanning mode [2,3,4], i.e., driven by smoothly adjusting its voltage. Adding amplifying mechanisms [5,6,7] for piezoelectric elements extends the stroke. Switching between scanning and stepping modes [8,9,10], the proposed device features mm-level working range at nm-level resolution. Referring to Figure 1, the most significant design factor is the parallel arrangement of two piezoelectric elements. Translational or rotational stepping motions are generated when this pair of piezoelectric elements are driven by a sawtooth waveform [11] in-phase or anti-phase, respectively. Our parallel design avoids serial stacking and achieves multiple DOF within a stiffer and more compact structure. This is an important feature for precision nano-positioners, in order to reduce vibration and thermal expansion. In brief, stepping mode offers a theoretically infinite stroke with µm-level resolution; scanning mode offers several µm-level stroke with nm-level resolution. Bridging two modes in 2-DOF is the goal of this study. The proposed system is designed to achieve several mm-level stroke with sub-µm resolution. If a higher accuracy is expected, the device can be switched to scanning mode, which has been well investigated in [2].

The stick-slip principle [12,13,14] is the foundation of the proposed nano-stepping motor. The periodic high slew-rate sawtooth waveform drives piezoelectric elements to overcome the friction force. Therefore, the inertia motor [15,16], or so-called impact drive [17,18], accumulates a series of steps forward. However, the step size does not remain consistent, because the friction force varies due to the microscopic nature of the contacting surfaces. Since open loop control is impossible, a standard method to control 1-DOF inertia in stepping motors is adding displacement sensors to form a feedback system. In the case of 2-DOF, crosstalk or coupling may occur between sensors if they are not aligned properly. This article proposes an optically visual servo control system to solve the afore-mentioned problem. Similar piezoelectric motors are used to manipulate a phase plate [19] in a transmission electron microscope (TEM). This requires high resolution, long stroke, and does not allow the electromagnetic field to interfere with electron beams. TEM itself is a perfect 2-DOF motion sensor that can navigate the piezoelectric motor accurately, but only inside TEM itself. This research work extends the nano-stepping motor’s applicability beyond the confines of a TEM. The instrumentation, image processing, control method, and validity are discussed in detail in the following sections. The experiments show that the proposed system functions effectively. 

## 2. Instrumentation and Control

### 2.1. Microscopic Imaging System

**Figure 2:**
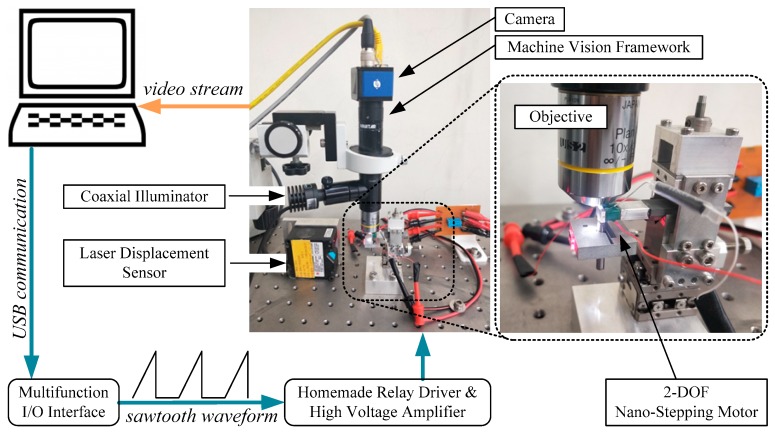
Block diagram and the photograph of the proposed visual servo control system for a piezoelectric 2-DOF nano-stepping motor. The camera acquires microscopic motion video and feeds it into an industrial computer. The image processing and motion control program drive the motor via the multifunction I/O interface and the high voltage amplifier.

Based on our proposed nano-stepping motor [1], a microscopic imaging system is installed as in Figure 2. The camera (eco655CVGE, SVS-Vistek, Seefeld, Germany) has 2448 × 2050 pixels. Central 1000 × 1000 pixels are cropped for the following study. Two microscopic objectives (Plan 4× and 10×, Olympus) have resolutions of 1 µm/pixel and 0.38 µm/pixel, respectively. The relation between the physical distance and the image pixel count is correlated by an accurate laser displacement sensor (LK-H020, Keyence, Osaka, Japan), which has a 20 nm repeatability. The laser displacement sensor is not used in the following experiments because it only has 1-DOF and cannot measure rotation. The machine vision framework (Precise Eye 1-6044, Navitar, San Ramon, CA, USA) connects the camera and the objective together. The framework’s coaxial illuminator provides suitable brightness for acquired images. The video stream is fed into an industrial computer (3.3 GHz, Intel i5 CPU) via Ethernet interface, and is then handled by LabVIEW (National Instrument, Austin, TX, USA). After image processing and decision making, the control signals are generated by a multifunction I/O interface (USB-6341, National Instrument, Austin, TX, USA), which provides two fast analog outputs for generating steep saw-tooth waveform, and at least one digital output for switching the relay. The state of the relay decides whether the sawtooth waveform pair is in-phase for translation, or in anti-phase for rotation. 

**Figure 3:**
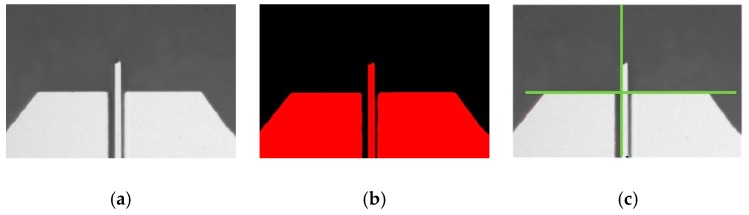
(**a**) The original, (**b**) the binarized, and (**c**) the edge-detected images of an AFM probe, which is treated as a micro marker of the proposed system. The width of above images is 1 mm.

An atomic force microscope (AFM) [20] probe is small, lightweight, and can serve as a micromachining tool bit [21,22,23]. Therefore, an AFM probe (Tap300Al-G, BudgetSenors, Sofia, Bulgaria) is glued to the rotor as the marker for micro vision, as labeled in Figure 1. The original image of the AFM probe is acquired as Figure 3a, and then binarized into Figure 3b. Our image processing program detects the edges, which are drawn as green lines in Figure 3c. The intersection of the crossed green lines is defined as the rotor’s position, the subject to be controlled in this research work.

### 2.2. Control System

As investigated, the proposed nano-stepping motor works up to 300 Hz but its velocity is not linear versus the driving frequency [1]. This phenomenon implies a suitable frequency range. After observing its stepping behavior under a microscope, we find that the device works smoothly between 70–90 Hz. Therefore, the driving frequency 80 Hz (i.e., 12.5 ms per sawtooth waveform) is fixed in the following experiments. For every task, the desired trajectory is separated into a series of checkpoints from start to finish. The amount of checkpoints depends on the intended precision and total length of the desired trajectory.

**Figure 4:**
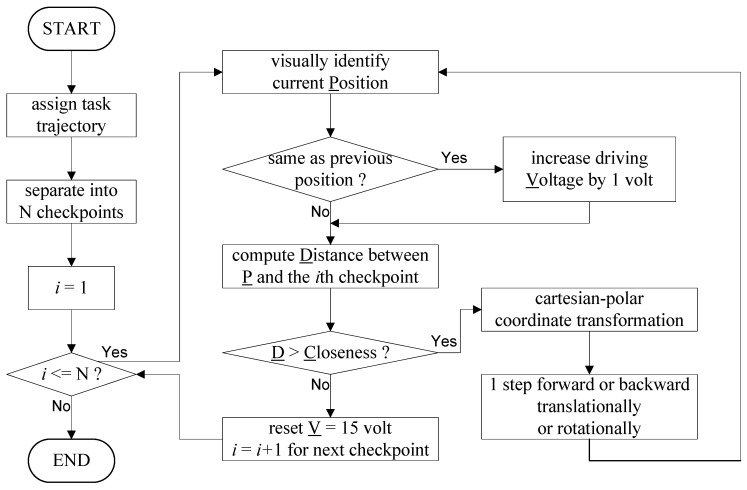
The control flow diagram of the control program. The goal is to trace a series of checkpoints. The driving voltage is increased gradually if the rotor is stuck.

Referring to Figure 4, we have developed a motion control program to let the rotor’s position to sequentially trace the checkpoints. Whenever the rotor’s position gets close enough to the current checkpoint, the control program commands the motor to trace the next checkpoint until the whole task is finished. Combining forward/backward with translation/rotation, there are four decisions that can be made. After Cartesian–polar coordinate transformation, the control program calculates its decision related to the current checkpoint, and then moves the motor step by step. If the current position is identical to the previous one, the driving voltage is increased gradually to overcome the local friction force. The driving voltage range is 15 V_pp_ to 30 V_pp_ [1]. The control program dynamically adjusts the “minimum walkable voltage”, which achieves precise steps without being stuck. The “closeness” value determines how closely the desired trajectory should be followed. Lower closeness values lead to less errors but take a longer time. The control system has to sacrifice accuracy for speed. The effect will be discussed in the following sections.

## 3. Experimental Result

The four-time and 10-time microscope objectives create fields of view (FOV) of 1 × 1 mm^2^ and 0.38 × 38 mm^2^, respectively. Three kinds of motion trajectories—straight lines, circles, and pentagrams—are demonstrated in this section.

### 3.1. Straight Line Trajectory

#### 3.1.1. Objective of Four-Time Magnification

**Figure 5:**
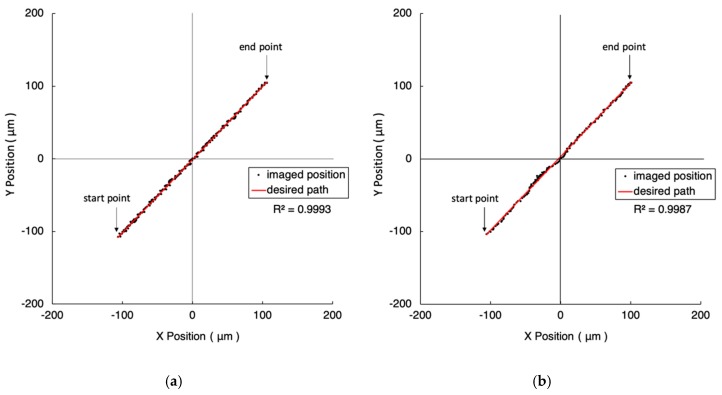
The experiment results of 300 μm straight line trajectories under the FOV of four-time objective. The closeness values are 1 µm and 5 µm in (**a**) and (**b**), respectively. The red line represents the desired path. The black dots are the imaged position of the marker driven by the 2-DOF nano-stepping motor.

As illustrated in Figure 5, the task trajectory is from the third quadrant to the first quadrant with a distance of 300 μm. Once the imaged position deviates from the desired path, the visual servo system corrects it back. Figure 5a,b shows the closeness values of one pixel (i.e., 1 µm) and five pixels (i.e., 5 µm), respectively. In Figure 5a, the coefficient of determination, the straightness, and the consumed time are 0.9993, 1.184 µm, 165 s, respectively. In Figure 5b, the coefficient of determination (COD), the straightness, and the consumed time are 0.9987, 1.506 µm, 108 s. The results reflect the trade-off between accuracy and speed.

**Figure 6:**
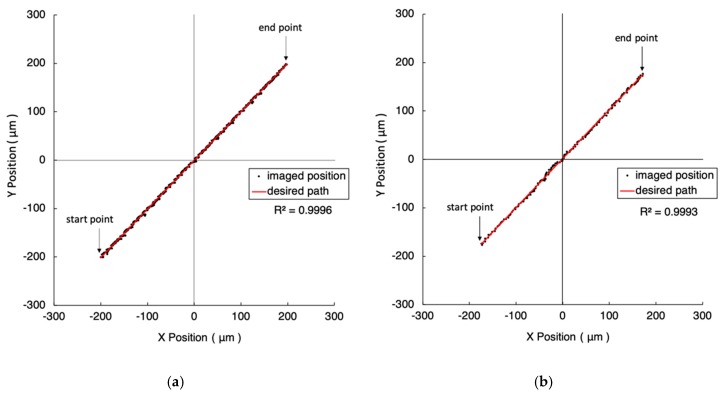
The experiment results of 500 μm straight line trajectories under the FOV of four-time objective. The closeness values are 1 µm and 5 µm in (**a**) and (**b**), respectively. The red line represents the desired path. The black dots are the imaged position of the marker driven by the 2-DOF nano-stepping motor.

Figure 6 shows the experimental results when the walking distance is extended to 500 μm, while other conditions are maintained. In Figure 6a, the coefficient of determination, the straightness, and the consumed time are 0.9995, 1.579 µm, 278 s, respectively. In Figure 6b, COD, the straightness, and the consumed time are 0.9993, 1.812 µm, 117 s. The results present a similar trend to Figure 5.

#### 3.1.2. Objective of 10-Time Magnification

**Figure 7:**
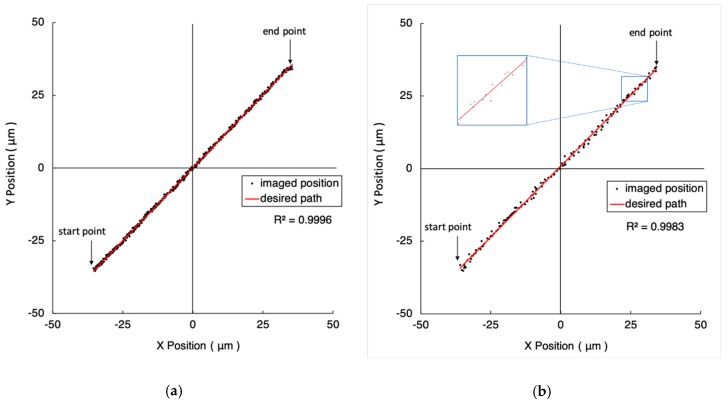
The experiment results of 100 μm straight line trajectories under the FOV of 10-time objective. The closeness values are 0.38 µm and 1.9 µm in (**a**) and (**b**), respectively. The red line represents the desired path. The black dots are the imaged position of the marker.

**Figure 8:**
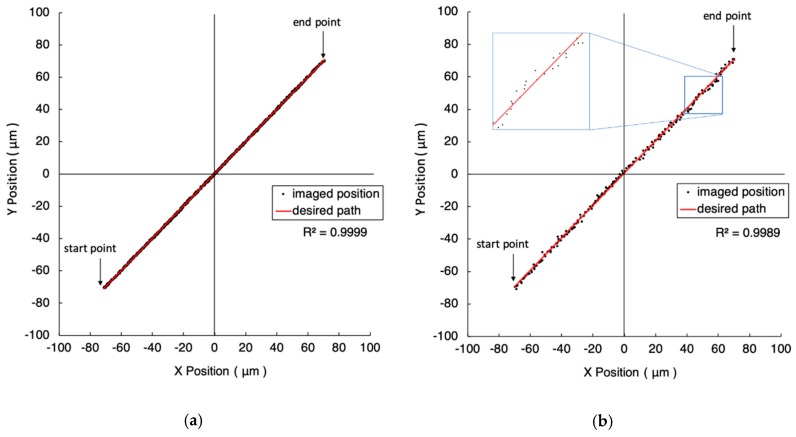
The experiment results of 200 μm straight line trajectories under the FOV of 10-time objective. The closeness values are 0.38 µm and 1.9 µm in (**a**) and (**b**), respectively. The red line represents the desired path. The black dots are the imaged position of the marker.

Replacing the microscope objective with a higher magnification enhances the image resolution and improves the motion accuracy. As illustrated in Figure 7 and Figure 8, the distances from their start points to end points are 100 μm and 200 μm, respectively. Figure 7a and Figure 8a show the results of closeness values of one pixel (i.e., 0.38 µm). The closeness values are five pixels (i.e., 1.9 µm) in Figure 7b and Figure 8b. The detailed experimental results are listed in Table 1.

### 3.2. Circle Trajectory

#### 3.2.1. Objective of Four-Time Magnification

**Figure 9:**
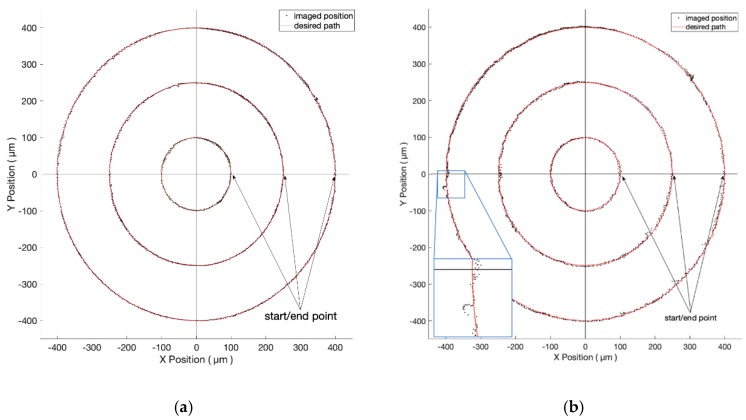
The experiment results of circles of different diameters under the FOV of four-time objective. The closeness values are 1 µm and 5 µm in (**a**) and (**b**), respectively.

As illustrated in Figure 9, the desired circular trajectories have diameters of 200 μm, 500 μm, and 800 μm. Figure 9a,b shows the closeness values of one pixel (i.e., 1 µm) and five pixels (i.e., 5 µm), respectively. The roundness varies from 7 µm to 27 µm. The time consumption varies from 174 s to 856 s. The detailed results are listed in Table 2. Obviously, lower closeness values lead to higher accuracy but more time is needed to complete the tasks.

#### 3.2.2. Objective of 10-Time Magnification

**Figure 10:**
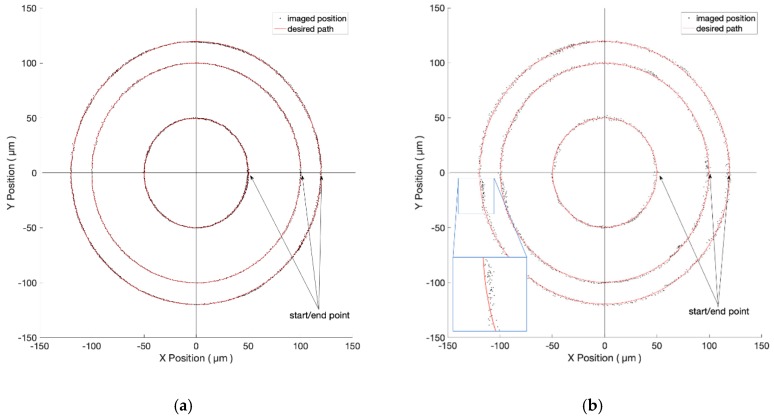
The experiment results of circles of different diameters under the FOV of 10-time objective. The closeness values are 0.38 µm and 1.9 µm in (**a**) and (**b**), respectively.

As described in Section 3.1.2, we now change to a higher magnification objective to achieve an improved image resolution. Theoretically, using higher magnification achieves better resolution but smaller FOV, which limits the full working range. Referring to Figure 10, the desired trajectories are circles of diameters at 100 μm, 200 μm, and 240 μm. Figure 10a,b shows the closeness values of one pixel (i.e., 0.38 µm) and five pixels (i.e., 1.9 µm), respectively. Table 2 indicates the detailed results. 

### 3.3. Pentagram Trajectory

**Figure 11:**
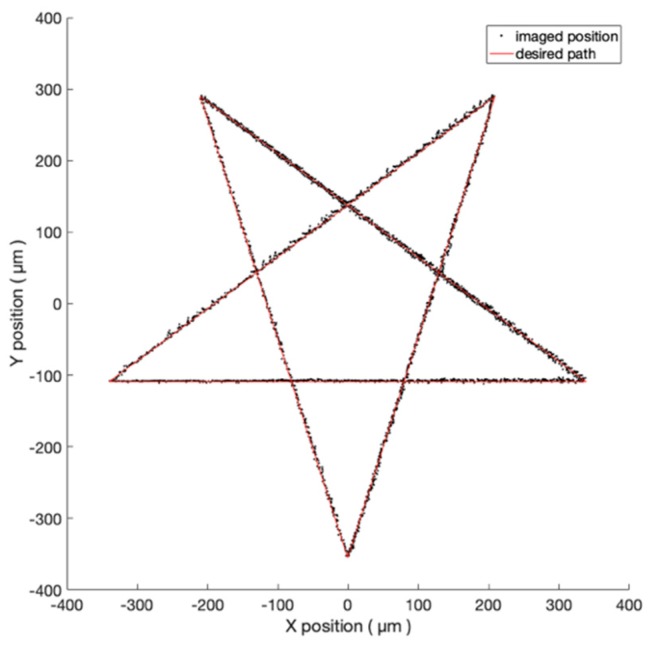
The experiment result of the pentagram trajectory under the FOV of four-time objective.

In addition to regular shapes, the proposed visual servo system has the ability to navigate the 2-DOF nano-stepping motor to walk along any other arbitrary trajectory, once it is defined by a series of checkpoints. An experiment of the pentagram trajectory is demonstrated in Figure 11 and recorded in a time-lapse microscopic video (see Supplementary Video) [24]. The diameter of its circumscribed circle is 700 μm. Five-thousand checkpoints are used. The averaged coefficient of determination, straightness, and the consumed times are 0.9994, 1.594 µm, 2310 s. 

## 4. Discussion

The experimental data of straight and circular trajectories are rearranged in Table 1 and Table 2, respectively. Higher magnification leads to a better resolution but a smaller working range. The COD, straightness, and roundness all indicate the precision of the controlled motion. All COD values of 45° linear motions are approaching 0.999, which means that the controller can compensate for the crosstalk between two DOFs effectively.

**Figure 12:**
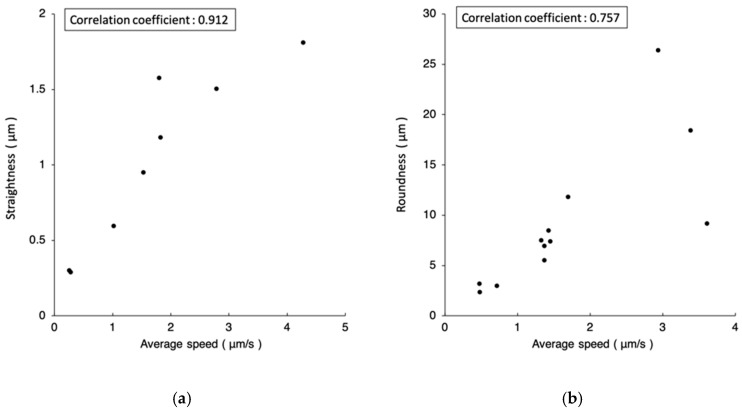
(**a**) Relation between the average speed and the straightness of linear motions in Table 1. (**b**) Relation between the average speed and the roundness of circular motions in Table 2. Positive correlation can be found between the error and the speed.

The smallest straightness 0.291 µm in Table 1 and roundness 2.380 µm in Table 2 both occur at the setting of 10-time objective and one-pixel closeness. The fastest speeds, 4.274 µm/s in Table 1 and 3.607 µm/s in Table 2, both occur at the setting of four-time objective and five-pixel closeness. Referring to Figure 12, plotting straightness/roundness versus the average speed, an obvious correlation can be found between the error and the speed.

**Figure 13:**
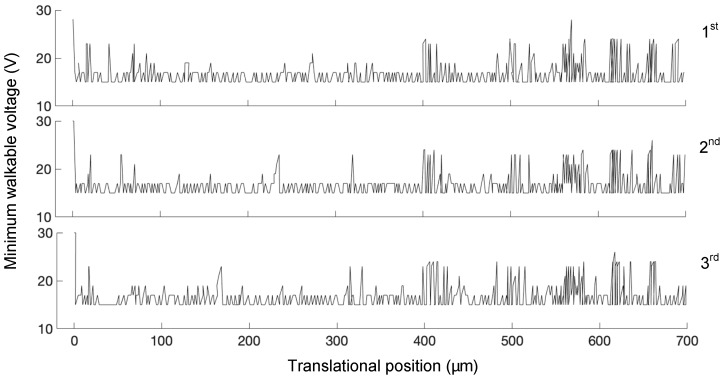
Minimum walkable voltage of 700 µm linear translational motion for three repeated tests.

After analyzing the experimental data, we found that “minimum walkable voltage” is position-relative and time-varying. Figure 13 shows the minimum walkable voltage of 700 µm linear translational motion for three repeated tests. Around 420 µm, the local friction force is greater than average; therefore, a higher voltage is needed to keep going forward. The results are similar, however, not identical over three tests because the contact condition had been changed over time. There does not exists a globally consistent minimum walkable voltage value, although the contact surfaces had been carefully polished. In summary, dynamically adjusting the driving voltage is a practical method to deal with the changing friction force.

As the voltage is increased, the rotor suddenly overcomes the maximum static friction and has the chance to step away from the desired trajectory. This phenomenon is illustrated in the zoomed screens of Figure 9b and Figure 10b. At those places with discontinuous friction force, the tracking error becomes greater than average. Eventually, our visual servo system can pull it back and reduces the error effectively.

Depending on the demands of a specific task, a balance between performance and budget is to be expected. Both the camera’s pixel density and the objective’s magnification affect precision. On the other hand, the camera’s frame rate and the computer’s image processing ability help to achieve higher speed. The direction of our future research is to build a coaxial multi-camera visual servo system. The large FOV image navigates the coarse motion quickly. The small FOV image controls the fine motion at a relatively low speed. The scheduling method between coarse and fine motions will release the power of the proposed 2-DOF nano-stepping motor. The third DOF, vertical to the image plane, will also be added. This will let the AFM probe fabricate microstructures.

## 5. Conclusions

The proposed 2-DOF nano-stepping motor offers the advantage of endless rotation and 6-mm translational stroke, at the cost of providing a consistent step size, making it impossible to use an open loop controller. The direct visual measurement is the most practical method to sense 360° rotation precisely and without contact. As a result, this article proposes a visual servo controller for the 2-DOF nano-stepping motor. The experiments show that the proposed controller can precisely navigate the rotor along various trajectories. In the case of following a 45° straight line, the best straightness is 0.291 µm at a speed of 0.273 µm/s. A higher speed could be achieved if a slightly greater tracking error (i.e., closeness) were acceptable. The trade-off between accuracy and speed remains inevitable. 

## Figures and Tables

**Table 1:** Experiment data of straight trajectories.

Objective	Task Distance (µm)	Closeness	COD	Straightness (µm)	Time (s)	AverageSpeed (µm/s)
four-time	300 µm	One pixel	0.9993	1.184	165	1.817
		Five pixels	0.9987	1.506	108	2.780
	500 µm	One pixel	0.9995	1.579	278	1.797
		Five pixels	0.9993	1.812	117	4.274 **
10-time	100 µm	One pixel	0.9996	0.291 *	366	0.273
		Five pixels	0.9983	0.597	99	1.012
	200 µm	One pixel	0.9999	0.303	787	0.254
		Five pixels	0.9989	0.952	131	1.523

* The condition of smallest error; ** the condition of fastest speed.

**Table 2:** Experiment data of circular trajectories.

Objective	Task Diameter (µm)	Closeness	Roundness (µm)	Time (s)	AverageSpeed (µm/s)
four-time	200	One pixel	7.516	475	1.322
		Five pixels	9.178	174	3.607 **
	500	One pixel	8.515	1104	1.423
		Five pixels	18.444	465	3.380
	800	One pixel	11.825	1482	1.696
		Five pixels	26.422	856	2.936
10-time	100	One pixel	2.380 *	659	0.477
		Five pixels	5.558	230	1.369
	200	One pixel	3.000	879	0.714
		Five pixels	6.965	460	1.367
	240	One pixel	3.222	1588	0.475
		Five pixels	7.430	521	1.447

* The condition of smallest error; ** the condition of fastest speed.

## Supplementary Materials

The following are available online at https://www.mdpi.com/2072-666X/10/12/811/s1, **Video:** The time-lapse microscopic video of the pentagram trajectory experiment.
